# A Three-Dimensional Finite Element Analysis Model of SAW Torque Sensor with Multilayer Structure

**DOI:** 10.3390/s22072600

**Published:** 2022-03-29

**Authors:** Zhipeng Li, Xu Meng, Bonan Wang, Chao Zhang

**Affiliations:** School of Transportation, Northeast Forestry University, Harbin 150040, China; lizp-nefu@nefu.edu.cn (Z.L.); linyedaxue@nefu.edu.cn (B.W.); chaozhang@nefu.edu.cn (C.Z.)

**Keywords:** surface acoustic wave, multi-layer structure, high frequency, finite element analysis, torque sensor

## Abstract

A three-dimensional finite element analysis model of surface acoustic wave (SAW) torque sensor based on multilayer structure is proposed in this paper. Compared with the traditional saw torque sensor with quartz as piezoelectric substrate, the SAW torque sensor with multilayer structure has the advantages of fast propagation speed and high characteristic frequency. It is a very promising torque sensor, but there is very little related research. In order to successfully develop the sensor, it is essential to understand the propagation characteristics and torque sensing mode of SAW in multilayer structure. Therefore, in this study, we first established a multi-layered finite element analysis model of SAW device based on IDT/128° Y-X lithium niobate/diamond/Si (100). Then, the effects of different film thicknesses on the characteristic frequency, electromechanical coupling coefficient, s parameter, and mechanical quality factor of SAW device without changing the wavelength are analyzed. Then, based on the finite element analysis, a three-dimensional research model of a new SAW torque sensor suitable for small diameter torsion bar (d = 10 mm) is established, and the relationship between saw device deformation and torque under the condition of small torque (±40 Nm) is tested. The shape variable is introduced into the finite element analysis model of multi-layer SAW device. Finally, the relationship between saw torque sensor with multi-layer structure and torque is established by using the deformation relationship, which shows the perfect curve of sensor performance.

## 1. Introduction

Among the emerging torque measurement principles and methods, torque measurement technology based on surface acoustic wave (SAW) principles has become one of the most attractive research topic directions due to its potential advantages. First, SAW propagates along a solid surface, and its propagation velocity is four to five orders of magnitude lower than that of an electromagnetic wave, which is convenient for the introduction or extraction of signals at any point in the propagation. Second, SAW devices are usually fabricated with the semiconductor planar process, which is especially suitable for mass production. Third, SAW devices have stable performances, high reliability, and good repeatability. Low sensitivity to external environmental factors can be achieved by optimizing the cutting shape of piezoelectric materials. At present, SAW devices have been widely used as temperature sensors, humidity sensors, chemistry sensors, acceleration sensors, biology, and torque sensors [1,2,3,4,5,6,7,8,9].

According to the reported SAW torque sensor data [10,11,12,13,14,15,16,17,18], most of these sensors use quartz as a piezoelectric substrate material, and the characteristic frequency is around 433 MHz. Low-temperature sensitivity can be achieved by using the specific cut shape of the piezoelectric body. However, with the rapid development of the fifth generation (5G) technology, low and medium frequency SAW devices will not meet the needs of the market. Therefore, whether from the development of SAW technology itself or from the application environment of SAW devices, SAW devices are developing towards the direction of high frequency and high performance.

The research on high-frequency SAW devices generally starts based on one of two aspects. Either a finer and more reliable interdigital transducer (IDT) finger strip from the structure is pursued, or higher basic sound velocity from the material is pursued [19,20,21,22,23,24,25,26,27]. However, the thinner IDT finger is often accompanied by more stringent manufacturing technology and means, which leads to a sharp increase in cost. Moreover, the ability of interdigital fingers that are too fine to resist high-frequency vibrations will decrease, and the high temperature generated by the high-frequency mechanical vibration can easily cause the melting of interdigital fingers. Therefore, due to the conditions of existing manufacturing technology and equipment, a higher basis for sound velocity-based materials or new piezoelectric structures, which are the main methods for improving the operating frequency and performance of SAW devices, should be identified because they are the only feasible way forward at this stage.

A three-dimensional finite element analysis model of SAW torque sensor with multilayer structure is proposed in this paper. The sensor model is based on the characteristics of small shaft diameter and low resonance frequency of existing SAW torque sensors. The structure of small SAW torque sensor with elastic torsion shaft is studied by finite element method. The effects of different film thicknesses on the characteristic frequency, electromechanical coupling coefficient, s parameter, and mechanical quality factor of the sensor are analyzed. Finally, the characteristic frequency shift of the multi-layer saw torque sensor model under the action of −40~+40 Nm external torque is studied, and the torque sensitivity and linearity of the SAW torque sensor in the specified torque range are analyzed.

## 2. Principle of Surface Acoustic Wave Torque Measurement

**Figure 1:**
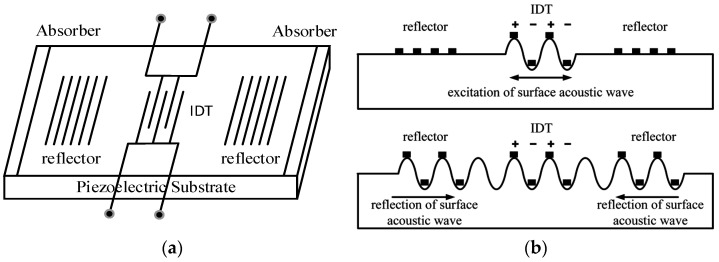
SAW device unit structure (**a**) one-port resonant type; (**b**) Propagation mode of SAW.

As shown in Figure 1, a one-port SAW resonator is the key component of SAW torque sensor. It is usually composed of piezoelectric substrate, IDT deposited on the surface of piezoelectric substrate, and reflection gratings on both sides. As the excitation element of SAW, the interdigital transducer is arranged on the piezoelectric substrate. It can convert the excitation signal into mechanical waves propagating along the surface of the piezoelectric substrate through the inverse piezoelectric effect. Reflectors are arranged on both sides of the piezoelectric substrate, which reflect the mechanical wave back to the interdigital transducer. The interdigital transducer uses the positive piezoelectric effect to convert the mechanical wave into electrical signal and transmit it to the receiver.

**Figure 2:**
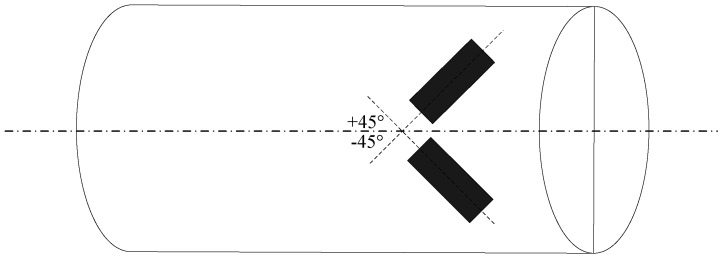
The schematic of the surface acoustic wave (SAW) torque sensor structure.

As shown in Figure 2, the saw torque sensor requires two SAW resonators with the same characteristic frequency to be installed vertically on the torsion bar shaft at an angle of ± 45° to the axis of the torsion bar. The differential arrangement is to eliminate the influence of temperature on the torque sensor. When the shaft is subjected to the action of torque M, the normal stress, σ, reaches the maximum value in the ± 45° direction, which is numerically equal to the maximum shear stress, $\tau_{\max}$, at this time. Therefore, the SAW resonator pasted along the ±45° direction mainly bears the effects of tension and compression. Numerically speaking, shear stress and normal stress can be expressed as Formulas (1) and (2):

(1) $$\tau_{\max}=\frac{16}{\pi D^{3}}M$$

(2) $$\sigma_{\pm 45^{o}}=\pm \frac{16\left(1+\mu \right)M}{\pi ED^{3}}$$

where, D is the diameter, M is the torque, μ is the Poisson’s ratio, and E is the elastic modulus.

Finally, by calculating the shear stress and normal stress, the expression of the relationship between torque and the characteristic frequency modification variable of SAW torque sensor can be obtained (3):

(3) $$M_{\pm 45^{o}}=\pm \frac{\Delta f\pi ED^{3}}{f_{0}16\left(1+\mu \right)\left(K^{\prime }-1\right)}$$

The center frequency of traditional SAW torque sensor is 433 MHz, which has the characteristics of small volume, good stability, passive wireless, and so on. However, there are still some problems, such as low characteristic frequency of resonator and unreasonable structure. (1) The size of a shaft body limits the installation mode. The minimum installation plane size of a sensor is a fixed value. With a decrease in the shaft body size (diameter), the depth of the cutting plane must be increased. Theoretically, when the diameter is smaller than the installation plane size, the sensor will not be installed, which is one of the fundamental reasons why a SAW torque sensor cannot be used for torque measurement of a small-sized flexible shaft. (2) The cutting plane on the shaft body for rotating work destroys the original structure of the shaft body, which inevitably leads to a stress concentration. A shaft body can easily fracture under the working conditions of a high torque or high speed, and this damage will be aggravated with a decrease in the shaft size. (3) It has been proved that different bonding methods, different adhesive materials, different adhesive thickness and different bonding angle will affect the characteristic frequency of SAW devices. Therefore, it is very difficult to keep these parameters unchanged during the experiment. (4) In recent years, due to the relatively mature research on quartz material, the research on SAW torque sensor has changed from device structure and substrate material to signal acquisition and processing. However, with the emergence and popularization of 5G communication technology, high frequency and over frequency SAW devices will gradually replace the existing SAW devices in the market position.

## 3. Simulation Model

### 3.1. Modeling a High Frequency SAW Resonator with a Layered Structure

The traditional SAW resonator is composed of piezoelectric substrate, interdigital transducer, reflective grating, and sound absorption strip. When the wavelength of incident wave is the same as that of reflected wave, SAW propagates in the form of standing wave between transducer and grating. The frequency of standing wave is the characteristic frequency of SAW device. The working principle of multi-layer SAW resonator is the same as that of traditional SAW resonator, but the difference lies in the structure of the device. The multi-layer SAW resonator does not use piezoelectric single crystal as the piezoelectric substrate, but uses a variety of piezoelectric thin film materials, even non-piezoelectric thin film materials superimposed to form a piezoelectric substrate.

In recent years, SAW devices with multi-layer structure have been widely concerned and studied. This is because the advantages and characteristics of different materials can be displayed by selecting different materials to form layered media. This is not only conducive to improving the performance of SAW devices, reducing the size of devices and reducing the cost, but can also develop new devices and sensors and broaden the application Field and scope. For example, a piezoelectric film may be coated on a non-piezoelectric material having a high sound velocity to increase the sound velocity of the device. The electromechanical coupling coefficient of the device can be improved by covering the material with high electromechanical coupling coefficient on the surface of the material with low electromechanical coupling coefficient. The device with zero temperature coefficient can be obtained by covering the material with negative temperature coefficient with positive temperature coefficient piezoelectric film. However, the multi-layer structure greatly increases the complexity of SAW device performance calculation, but also reduces the accuracy. The finite element method (FEM) can improve the simulation accuracy and reduce the calculation time. In this study, COMSOL multiphysics 5.6 finite element analysis software is used for simulation research.

**Figure 3:**
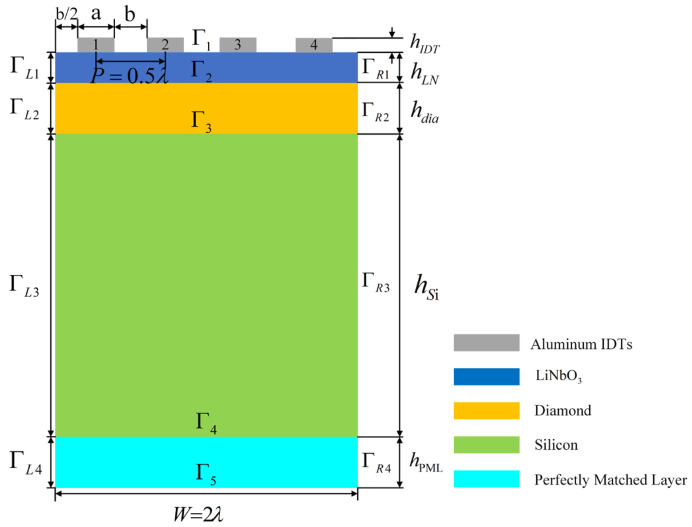
Two-dimensional simulation model of SAW resonator with layered structure.

The 2D pattern of SAW resonator for simulation is shown in Figure 3. The initial values of the material and structure dimensions of each layer in the X_3_ direction were as follows: perfectly matched layer (pml), a silicon substrate (matrix layer), diamond film layer (growth layer), LiNbO_3_ film layer (piezoelectric layer), and aluminum electrode (interdigital transducer). See Table 1, Table 2, Table 3, Table 4 and Table 5 for material parameters, structural parameters, boundary conditions, and electrode polarity of all materials.

### 3.2. Establishment of the Torque Sensor Model

To overcome the problems mentioned in Section 2 and to match the steering shaft with a smaller diameter, a new SAW torque sensor was proposed in this study. The new SAW torque sensor adopted a symmetrical structure that was composed of a restraint seat, support plane, PCB circuit board, and SAW resonator. The support and restraint seats and the support plane were integral structures, and the support plane was square. The support and restraint seats were pressed onto the elastic axis with a steel belt. A SAW resonator ±45° was welded onto the PCB board through pins and the PCB board was pasted onto the support plane.

**Figure 4:**
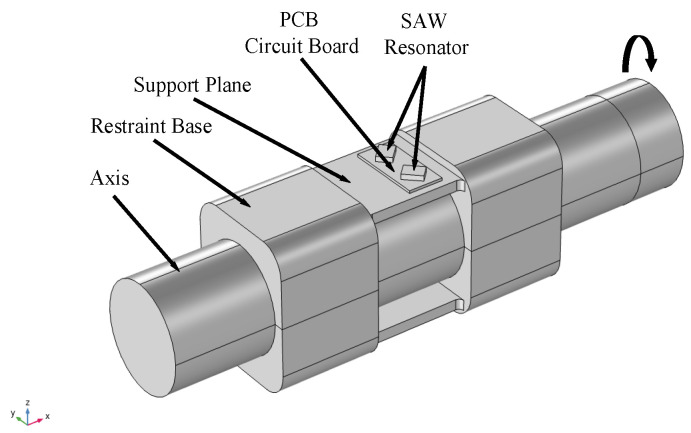
3D schematic diagram of new SAW torque sensor.

The 3D structure of the SAW torque sensor for simulation is shown in Figure 4. The diameter of the flexible shaft was set to be 10 mm. In the solid mechanics node, one end of the rigid axis was set as a fixed constraint, and the other end was set in the direction of the rigid domain force resistance-y.

## 4. Results and Discussion

### 4.1. Analysis of the SAW Propagation Characteristics of the SAW Resonator with a Layered Structure

**Figure 5:**
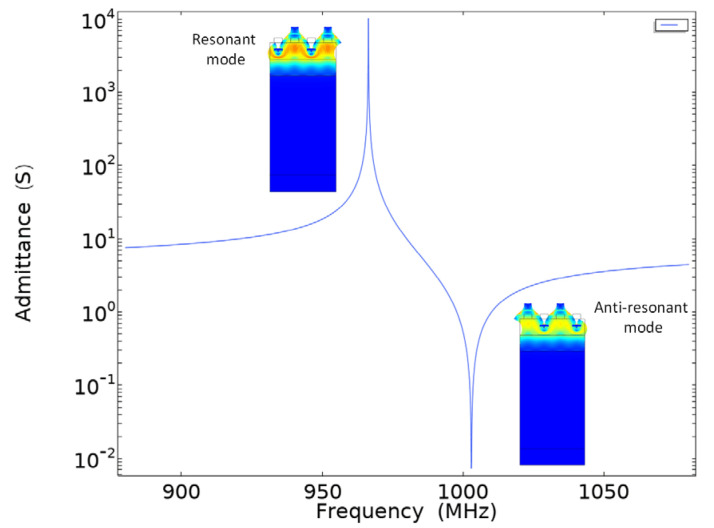
Admittance curve under initial conditions.

The admittance curve of layered SAW resonator under initial conditions is shown in Figure 5. The illustration in Figure 5 shows the deformed shape when SAW is excited. When SAW resonators function, there are two characteristic frequencies for short circuit conditions, which are called the resonant and anti-resonant frequency.

**Figure 6:**
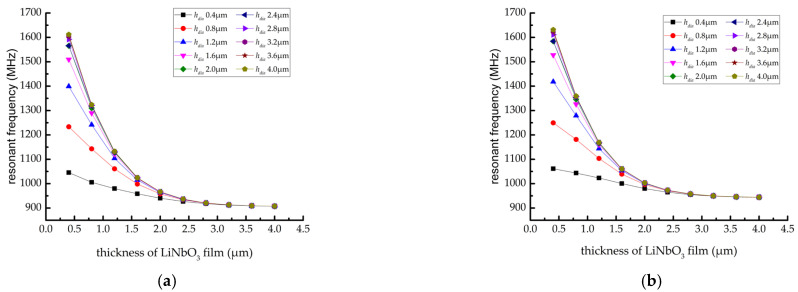
Variation curve of resonant and anti-resonant frequency (**a**) resonant frequency and (**b**) anti-resonant frequency.

Through the simulation of the SAW resonator model with the multi-layer structure mentioned above, the positive and anti-resonant frequency variation curves were obtained, as shown in Figure 6. The results show that: (1) the thickness of diamond film $h_{dia}$ is unchanged, and the LiNbO_3_ film thickness is $h_{LN}>2.4\;\mu m\;\left(0.6\lambda \right)$; the resonant and anti- resonant frequency of SAW resonator no longer change significantly. When LiNbO_3_ film thickness $h_{LN}<2.4\;\mu m\;\left(0.6\lambda \right)$, it can be found that the resonant and anti- resonant frequency of SAW resonator increase significantly with the decrease in LiNbO_3_ film thickness $h_{LN}$. (2) When LiNbO_3_ film thickness $h_{LN}<1.2\;\mu m\;\left(0.3\lambda \right)$, and the diamond film thickness $h_{dia}$ from 0.4 μm 0.1λ Increase to 1.6 μm 0.4λ, the resonant and anti- resonant frequency of SAW resonators are obviously mentioned. When the diamond film thickness $h_{dia}$ increases upward, the increase in resonant and anti- resonant frequency of SAW resonators tends to be flat.

Electromechanical coupling coefficient (K^2^) represents the coupling degree between mechanical energy and electrical energy of piezoelectric body, and is an important physical quantity to measure the piezoelectric strength of piezoelectric materials. Based on finite element simulation, the electromechanical coupling coefficient can be defined according to the relative interval between the resonant and anti-resonant frequency shown in Formula (4) [28].

(4) $$K^{2}=\left(\frac{\pi^{2}}{4}\right)\left(\frac{f_{m-}-f_{m+}}{f_{m-}}\right)$$

where, $f_{m+}$ is the resonant frequency and $f_{m-}$ is the anti-resonant frequency.

**Figure 7:**
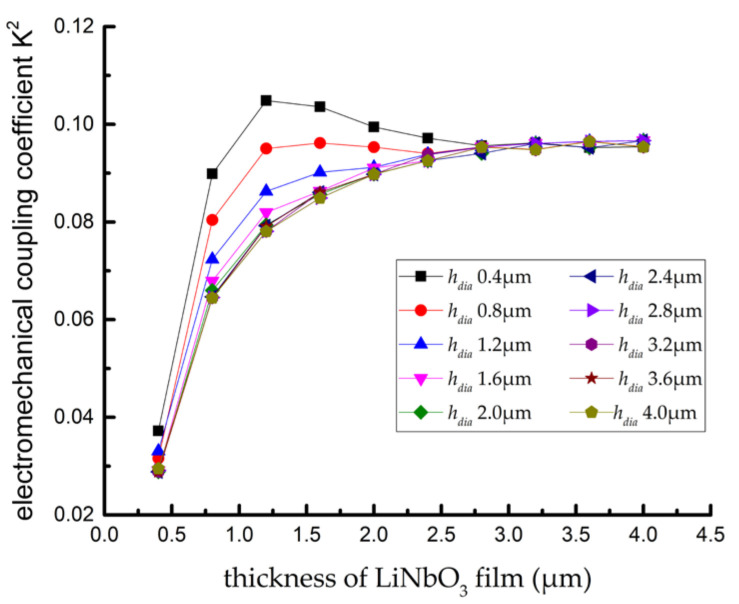
Distribution curve of electromechanical coupling coefficient K^2^.

The electromechanical coupling coefficient of the SAW resonator with a multi-layer structure for different film thickness conditions could be calculated, as shown in Figure 7. The results show that: (1) when $h_{dia}>1.6\;\mu m\;0.4\lambda$, no matter how $h_{LN}$ changes, the variation law of electromechanical coupling coefficient (K^2^) of SAW resonator remains the same, that is, with the decrease in $h_{LN}$, the electromechanical coupling coefficient (K^2^) gradually decreases from 0.98 to the minimum value of 0.029; (2) When $h_{dia}<1.6\;\mu m\;0.4\lambda$, with the decrease in $h_{LN}$, the electromechanical coupling coefficient (K^2^) will experience a process of slowly decreasing, then increasing, and, finally, rapidly decreasing. When $h_{dia}=0.4\;\mu m\;0.1\lambda$ and $h_{LN}=1.2\;\mu m\;0.3\lambda$, the electromechanical coupling coefficient (K^2^) is taken as the maximum value of 0.105.

**Figure 8:**
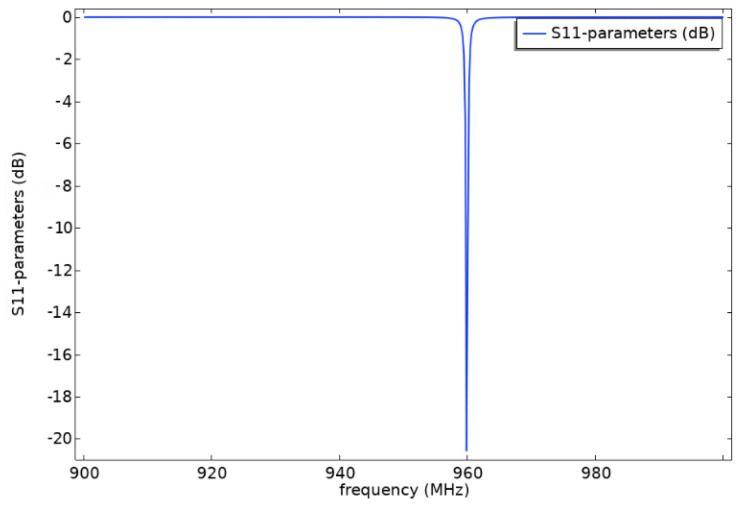
S-parameter analysis curve.

S parameter (scattering coefficient) is an important parameter to study the performance of SAW resonator. It reflects the relationship between incident power wave and reflected power wave of a network system. The network system includes two ports (port 1 and port 2), and the signal can be input or output by any one of them. According to different signal acquisition methods and ports, the general s parameters can be divided into S11, S22, S12, and S21. The performance of single port resonator is mainly evaluated according to its S11 parameter curve. From the smoothness of S11 parameter curve, the reflection coefficient of the resonator can be qualitatively analyzed. If the curve is smoother, the reflection coefficient of the resonator will be larger and its performance will be better. The S11 curve of SAW resonator and its reflection coefficient meet the following relationship:

(5) $$S11=20\log(1-\frac{W_{R}}{W_{I}})$$

where $W_{R}$ is the reflected power and $W_{I}$ is the incident power. According to the above formula, the larger the amplitude of the S11 parameter curve of the device, the higher the reflection coefficient of the device. Figure 8 shows the S11 parameter curve of multi-layered SAW resonator under the condition of initial structural parameters. From the figure, we can see that the multi-layered SAW resonator has a good response near 960 MHz, and the curve is smooth as a whole without side lobe. The amplitude is −20.5 dB and the frequency at the amplitude is 959.6 MHz.

The sensor will lose a lot of energy in wireless communication with the reader. This loss is inevitable, and this loss will directly affect the wireless transmission distance. In order to ensure the wireless transmission distance between the sensor and the reader, it is necessary to improve the quality factor of the sensor terminal as much as possible. For the single port resonator with layered structure, the relationship between the quality factor $Q_{m}$ and the device structure is shown in Formula (6) [29]:

(6) $$Q_{m}=\frac{\pi L_{c}}{\lambda_{0}\left(1-\tanh_{LN}(\left|r_{s}\right|N_{g})\right)}$$

where $N_{g}$ is the number of short-circuit electrodes in the reflecting cavity, $r_{s}$ is the reflectivity of a single short-circuit electrode, and $L_{c}$ is the effective length of the resonant cavity.

**Figure 9:**
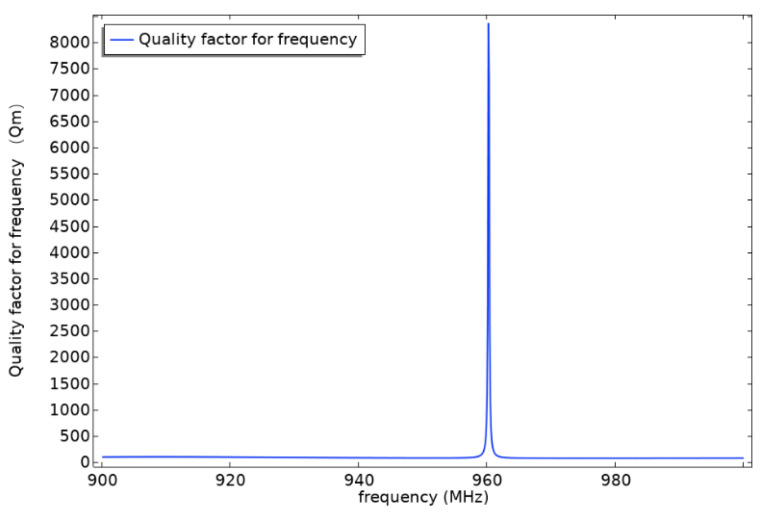
$Q_{m}$-parameter analysis curve.

Figure 9 shows the parameter curve of quality factor $Q_{m}$ of multi-layered SAW resonator under the condition of initial structural parameters. From the figure, we can see that the multi-layered SAW resonator has a good response near 960 MHz, and the curve is smooth as a whole without side lobe. The amplitude is 8370 and the frequency at the amplitude is 960.2 MHz.

### 4.2. Analysis of the SAW Torque Sensor with a Layered Structure

**Figure 10:**
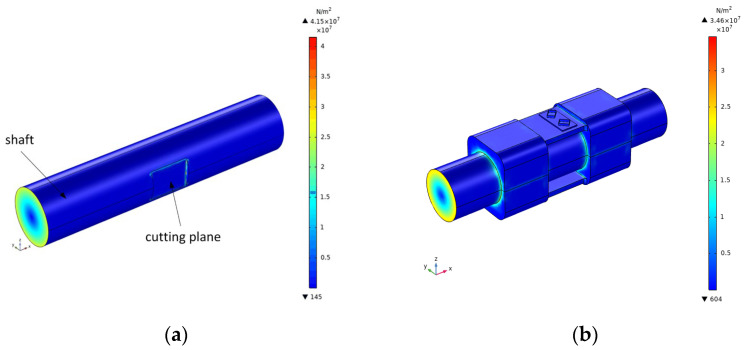
Surface shear stress cloud diagram of SAW torque sensor: (**a**) traditional forms and (**b**) new SAW torque sensor.

As shown in Figure 10, when the SAW torque sensor bore the action of 40 Nm torque, the surface shear stress reached the maximum at the contact between the inner surface of the restraint seat and the outer surface of the steering shaft due to the existence of the surface friction. Compared with the cutting plane SAW torque sensor, the surface shear stress was reduced by 17%, which improved the stability and safety of the axle body operation. Additionally, there was no stress concentration phenomenon between the steering shaft and the sensor mechanical structure, and the stress was more uniform.

**Figure 11:**
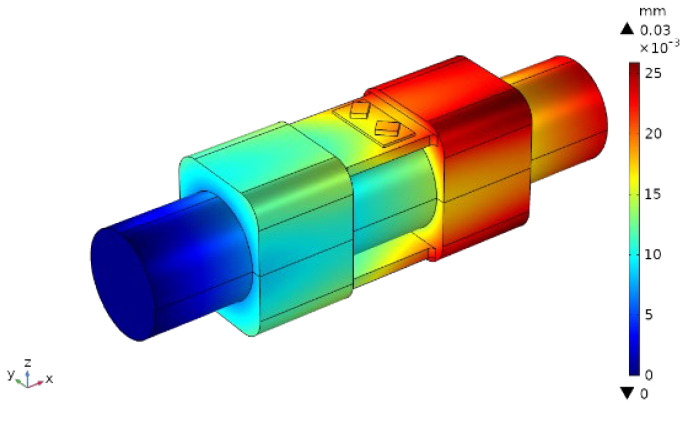
Cloud chart of total shape variable.

The displacement nephogram of the whole sensor is shown in Figure 11. The maximum displacement was located on the flexible axis and support seat near the driver’s hand input end, and the maximum deformation was $0.03\times 10^{-3}$ mm. However, according to the probe feedback data applied to the end faces of SAW resonator 1 and SAW resonator 2, for the same conditions, the shape variables of SAW resonators 1 and 2 arranged diagonally on the upper support plane were $0.0188\times 10^{-3}$ mm and $0.0189\times 10^{-3}$ mm, respectively. The deformations of SAW resonators 1 and 2 were almost the same when the torque occurred.

**Figure 12:**
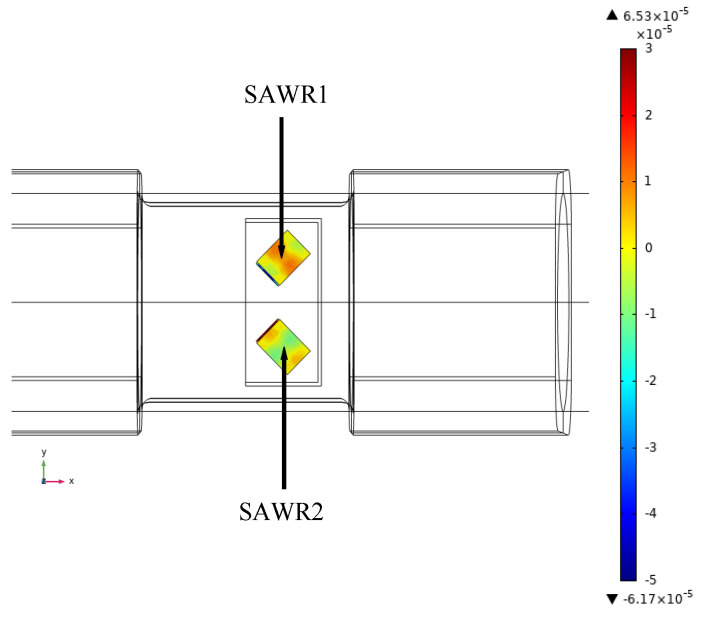
SAWR shape variable cloud.

As shown in Figure 12, the strain analysis of SAWR is carried out separately.

**Figure 13:**
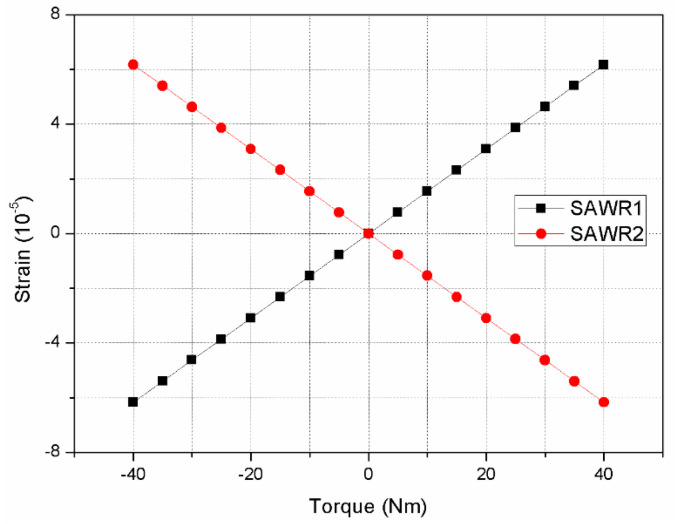
Strain curve of SAWR in the working range of −40~40 Nm torque.

When the torque of 40 Nm is applied to the rotating shaft in a counterclockwise direction, the phenomenon of accumulation from both sides to the middle of SAWR1 appears, which indicates that resonator 1 is compressed, and the maximum strain is $6.53\cdot 10^{-5}$. On the contrary, SAWR2 spreads from the middle to both sides, indicating that resonator 2 is under tension, and the maximum strain is $-6.17\cdot 10^{-5}$. In the full range (−40~+40 Nm), the variation curve of SAWR1 and SAWR2 strain with input torque is shown in Figure 13.

It was assumed that the SAW resonator would a produce corresponding strain s(M) under the action of the torque M. At that time, the propagation velocity and wavelength of the SAW would change accordingly to $v^{\prime }\left(M\right)=v_{0}+\Delta v(s(M))$ and $\lambda^{\prime }(M)=\lambda_{0}(1+s(M))$, respectively. Therefore, when the torque was applied, the operating frequency of the SAW resonator became:

(7) $$f_{0}{}^{\prime }=\frac{v^{\prime }\left(s(M)\right)}{\lambda^{\prime }\left(s(M)\right)}=\frac{v_{0}+\Delta v(s(M))}{\lambda_{0}(1+s(M))}=\frac{v_{0}+\Delta v(M)}{\lambda_{0}+\Delta \lambda (M)}=f^{\prime }(M)$$

According to Formula (7), the relationship between SAW resonator and torque can be established. The torque can be measured by detecting the working frequency of SAW resonator. Under ideal conditions, two identical SAW resonators, SAWR1 and SAWR2, are arranged according to the structure shown in Figure 13. In the process of automobile steering, the characteristic frequencies of SAWR1 and SAWR2 are affected not only by strain ∇ε in opposite direction, but also by the same environmental factors ∇δ (temperature, humidity, etc.). The mathematical expression is as follows:

(8) $$f_{SAWR1}=f(\varepsilon +\nabla \varepsilon ,\delta +\nabla \delta )$$

(9) $$f_{SAWR2}=f(\varepsilon -\nabla \varepsilon ,\delta +\nabla \delta )$$

Through the polynomial expansion of Formulas (8) and (9) (retaining the first two terms), and then subtracting, the following equation can be obtained:

(10) $$f_{SAWR1}-f_{SAWR2}=2\frac{\partial f}{\partial \varepsilon }\nabla \varepsilon +2\frac{\partial^{2}f}{\partial \varepsilon \partial \delta }(\nabla \varepsilon \nabla \delta )$$

Suppose that ∇ε and ∇δ are independent and linearly independent, so:

(11) $$2\frac{\partial^{2}f}{\partial \varepsilon \partial \delta }(\nabla \varepsilon \nabla \delta )=0$$

Therefore, the double SAW resonators for differential processing of output signal can be used to completely eliminate the influence of environmental factors on the sensor output signal, in theory, so that the sensitivity of torque measurement can be doubled. However, in practical application, the influence of environmental factors on the measurement structure cannot be completely eliminated. The main reasons are: (1) SAWR1 and SAWR2 cannot make exactly the same; (2) SAW torque sensor in the process of manufacture and installation, SAWR1 and SAWR2 cannot bear the same strain with opposite sign; (3) in order to get the ideal result, the calculation formula behind the polynomial is ignored.

**Figure 14:**
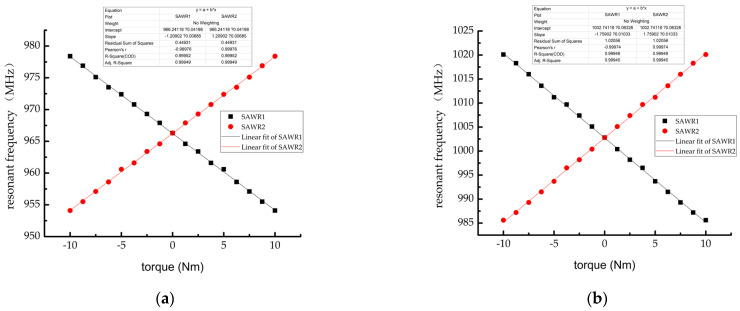
Variation curve of SAWR resonant frequency with sensor input torque (**a**) resonant frequency and (**b**) anti-resonant frequency.

After the strain data of the resonator was added to the simulation model of the layered SAW resonator, the corresponding relationship between the torque and the SAW resonant frequency was established; the relationship curve is shown in Figure 14. The results showed that the linearity of the sensor output signal was good within the range of the torque measurement, and the fitted linear equations were resonant frequency $f_{M+}=966.24118\pm 1.21x\;\mathrm{MHz}$ and anti-resonant frequency $f_{M-}=1002.74118\pm 1.76x\;\mathrm{MHz}$.

It should be noted that the above simulation results are analyzed in the ideal state; that is, the multi-layer structure has different shape variables under the action of external stress, the pasting angle error of SAW resonator and the hysteresis and loss phenomenon in the process of torque transmission are ignored. These influencing factors will inevitably have corresponding effects on the output frequency of the sensor, which need to be clarified in the follow-up research to reduce the gap between simulation analysis and experimental test as much as possible.

## 5. Conclusions

In this work, we propose a multi-layer structure simulation model of high frequency SAW torque sensor and carry out detailed simulation research in two main aspects. Firstly, for the multi-layer structure of IDT/128° Y-X lithium niobate/diamond/Si (100), the effects of working wavelength, LiNbO3 film thickness, and diamond film thickness on the resonant frequency, phase velocity, electromechanical coupling coefficient, s parameter, and mechanical quality factor of SAW resonator are analyzed. The simulation results show that the resonant frequency of the resonator increases with the decrease in the working wavelength. When the working wavelength is 4 μm, the maximum value of the electromechanical coupling coefficient appears at the wavelength of 0.3 times of LiNbO_3_ film thickness and 0.1 times of diamond film thickness. The maximum value is 0.105. Under the condition of initial structural parameters, the S parameter of multilayer SAW resonator is −20.5 dB and the quality factor $Q_{m}$ is 8370. With the increase in the thickness of LiNbO_3_ film, the SAW propagation in the layered structure is more similar to that in the LiNbO_3_ single crystal. On the contrary, with the decrease in the thickness of the LiNbO_3_ film, the SAW propagates more in the diamond film, and the SAW transmission in the layered structure is more similar to that in the LiNbO_3_ single crystal. Besides, the electromechanical coupling coefficient of SAW resonator increases with the decrease in the thickness of LiNbO_3_ film and diamond film. Secondly, aiming at the problem that the diameter of the elastic torsion shaft is too small, a new SAW torque sensor structure is studied, and the torque measurement principle under the new structure is analyzed. The simulation results show that the new saw torque sensor reduces the surface shear stress by 17% under the same torque, and the strain linearity of SAW resonator is good in the range of −40~40 Nm torque measurement. Finally, the relationship between the applied torque and the output frequency is analyzed, and the unconventional frequency output curve is obtained. The linearity is very good, which fully proves the high frequency SAW of layered structure; a good working performance of the torque sensor.

The research of this method is not limited to this, but can also be expanded, such as whether other thin-film materials or other layered structures can be used to achieve better SAW propagation characteristics; the influence of external conditions, such as temperature, humidity, and vibration, on the performance of saw torque sensor with a layered structure. In future work and research, the authors will make a sample of high frequency SAW torque sensor with the layered structure to demonstrate the method proposed in this work, and compare the performance of the high frequency saw torque sensor with the traditional saw torque sensor more clearly.

## Figures and Tables

**Table 1:** Non piezoelectric material parameters used in the simulation model.

Material	AI(IDT)	Diamond	Si(100) Substrate, PML
Density, ρ [kg/m^3^]	2700	3515	2329
Relative dielectric constant, $\varepsilon_{rs}$	1	5.1	11.7
Young’s modulus, E [Pa]	$70\times 10^{9}$	$105\times 10^{10}$	$170\times 10^{9}$
Poisson’s ratio, μ	0.33	0.1	0.28

**Table 2:** Parameters of piezoelectric materials used in the simulation model.

Material	128°Y-X LiNbO_3_			
Density, ρ [kg/m^3^]	4700			
Relative dielectric constant, $\varepsilon_{rs}$	$\varepsilon_{11}$	$\varepsilon_{22}$	$\varepsilon_{33}$	$\varepsilon_{23},\varepsilon_{32}$
	44	38.3144	34.6856	−7.2772
Coupling matrix, $e_{ES}$ [cm^−2^]	$e_{15}$	$e_{16}$	$e_{21}$	$e_{22}$
	4.4548	0.3079	−1.8496	4.1988
	$e_{23}$	$e_{24}$	$e_{31}$	$e_{32}$
	−1.9210	0.2205	1.6968	−2.9786
	$e_{33}$	$e_{34}$	-	-
	1.8334	0.2339	-	-
Elastic matrix, $C_{E}$ [10^11^ Pa]	$C_{11}$	$C_{22}$	$C_{33}$	$C_{44}$
	2.0300	1.9442	2.2205	0.7576
	$C_{55}$		$C_{12},C_{21}$	$C_{13},C_{31}$
	0.5695	0.7805	0.7707	0.5793
	$C_{14},C_{41}$	$C_{23},C_{32}$	$C_{24},\;C_{42}$	$C_{34},C_{43}$
	0.1285	0.9076	0.0967	0.0853
	$C_{56},C_{65}$	-	-	-
	−0.0510	-	-	-

**Table 3:** Initial structural parameters of simulation model.

Name	Parameter
λ	4 μm
a	0.25λ (1 μm)
b	0.25λ (1 μm)
P	0.5λ (1 μm)
W	2λ (8 μm)
$h_{IDT}$	0.15λ (0.6 μm)
$h_{LN}$	0.5λ (2 μm)
$h_{dia}$	0.5λ (2 μm)
$h_{Si}$	3λ (12 μm)
$h_{\mathrm{PML}}$	0.5λ (2 μm)

**Table 4:** Boundary conditions used in simulation models.

Boundary	Mechanical Conditions	Electrical Conditions
$\Gamma_{1}$	Free	Zero charge
$\Gamma_{2}$, $\Gamma_{3}$, $\Gamma_{4}$	Free	Continuity
$\Gamma_{5}$	Fixed	Ground
$\Gamma_{R1}$, $\Gamma_{R2}$, $\Gamma_{R3}$, $\Gamma_{R4}$, $\Gamma_{L1}$, $\Gamma_{L2}$, $\Gamma_{L3}$, $\Gamma_{L4}$	Periodic boundary conditions	Periodic boundary conditions

**Table 5:** Electrode polarity of IDT in simulation model.

Electrode Number	Electrode Polarity
1	+1 V
2	Grounding
3	+1 V
4	Grounding

## Data Availability

Not applicable.
